# Navigating relationship dynamics, pregnancy and fatherhood in the *Bukhali* trial: a qualitative study with men in Soweto, South Africa

**DOI:** 10.1186/s12889-023-17153-x

**Published:** 2023-11-08

**Authors:** Catherine E. Draper, Molebogeng Motlhatlhedi, Jackson Mabasa, Tshepang Headman, Sonja Klingberg, Michelle Pentecost, Stephen J. Lye, Shane A. Norris, Lukhanyo H. Nyati

**Affiliations:** 1 SAMRC/Wits Developmental Pathways for Health Research Unit, Department of Paediatrics, Faculty of Health Sciences, University of the Witwatersrand, Johannesburg, South Africa; 2 Department of Global Health and Social Medicine, King’s College London, London, UK; 3 Toronto and Departments of Obstetrics and Gynecology, Physiology and Medicine, Lunenfeld-Tanenbaum Research Institute, Sinai Health, University of Toronto, Toronto, ON Canada; 4 School of Human Development and Health, University of Southampton, Southampton, UK; 5 Interprofessional Education Unit, Faculty of Community and Health Sciences, University of the Western Cape, Cape Town, South Africa

**Keywords:** Fathers, LMIC, Qualitative

## Abstract

**Background:**

South Africa has a complex range of historical, social, political, and economic factors that have shaped fatherhood. In the context of the *Bukhali* randomised controlled trial with young women in Soweto, South Africa, a qualitative study was conducted with the male partners of young women who had become pregnant during the trial. This exploratory study aimed to explore individual perceptions around relationship dynamics, their partner’s pregnancy, and fatherhood of partners of young women in Soweto, South Africa.

**Methods:**

Individual, in-depth interviews were conducted with male partners (fathers, n = 19, 25–46 years old) of *Bukhali* participants. A thematic approach was taken to the descriptive and exploratory process of analysis, and three final themes and subthemes were identified: (1) relationship dynamics (nature of relationship, relationship challenges); (2) pregnancy (feelings about the pregnancy, effect of the pregnancy on their relationship, providing support during pregnancy; and 3) fatherhood (view of fatherhood, roles of fathers, influences on views and motivation, challenges of fatherhood).

**Results:**

While most male participants were in a committed (“serious”) relationship with their female partner, less than half of them were cohabiting. Most reported that their partner’s pregnancy was not planned, and shared mixed feelings about the pregnancy (e.g., happy, excited, shocked, nervous), although their views about fatherhood were overwhelmingly positive. Many were concerned about how they would economically provide for their child and partner, particularly those who were unemployed. Participants identified both general and specific ways in which they provided support for their partner, e.g., being present, co-attending antenatal check-ups, providing material resources. For many, the most challenging aspect of fatherhood was having to provide financially. They seemed to understand the level of responsibility expected of them as a father, and that their involvement and presence related to love for and connection with their child. Participants’ responses indicated that there were some changes in the norms around fatherhood, suggesting that there is a possibility for a shift in the fatherhood narrative in their context.

**Conclusions:**

These findings suggest that the complex array of factors influencing fatherhood in South Africa continue to play out in this generation, although promising changes are evident.

## Background

Developmental Origins of Health and Disease (DOHaD) research has manifested in a substantial number of studies on the potential effects of the health and lifestyle of mothers around the time of pregnancy on the health of their children. Consequently, a structural imbalance in DOHaD data exists in that there is much less paternal and postnatal data as compared to maternal data during pregnancy. This imbalance can be lessened by more good quality data of potential postnatal, partner and paternal effects [1, 2]. The important role of fathers and their provision of social support is increasingly being acknowledged in life course research, but it is recognised that there are numerous contextual factors that influence men’s involvement and ability to provide this support [3]. South Africa has a complex range of historical, social, political, and economic factors that have shaped fatherhood, in particular the discriminatory and socially disruptive effects of apartheid [4–7]. This country has one of the highest rates of father absence in Africa, [4] but father ‘absence’ is a contested term in South Africa, given its relationship to socio-economic and political contexts, [7, 8] and that there are fathers who do not reside with their children, but remain involved [4].

Although the role of fathers is not always clear, and norms around interactions with their children can be complicated and inconsistent in South Africa, [5] the importance of involvement of fathers in their children’s lives from an early age, starting in pregnancy, has been highlighted, including for maternal mental health, [9] and notably in low-income communities [10]. However, there are few published health interventions involving fathers in South Africa [11–14]. There appear to be some positive shifts in the narrative in South Africa about father involvement and traditionally gendered caregiving roles; [4–6, 15] this has been observed in other African countries as well [16, 17]. For example, research with expectant fathers in Senegal has also highlighted how Eurocentric notions of fathers’ involvement do not adequately capture contextually specific notions of involvement [18].

### Healthy life trajectories Initiative: *Bukhali* trial

The Healthy Life Trajectories Initiative (HeLTI) is an international consortium developed in partnership with the World Health Organization (WHO) in Canada, India, China, and South Africa. Drawing on interdisciplinary evidence from DOHaD, and adopting a life-course perspective, HeLTI hypothesises that an integrated complex intervention, comprising a continuum of care from preconception, through pregnancy, infancy and early childhood will promote young women’s physical and mental health, in order to establish healthier trajectories for themselves and future offspring. As part of HeLTI SA, the *Bukhali* randomised controlled trial is being conducted with 18–28-year-old women in Soweto, [19] a densely populated, predominantly low-income, urban, and multilingual setting in Johannesburg.

Extensive qualitative work has been (and is being) conducted as part of the *Bukhali* trial process evaluation to characterise the lived experiences of the trial participants [20, 21]. The social support experienced by these women has been explored, highlighting the importance of social support from their partners, and particularly the father of their baby if they have become pregnant [21]. In work currently under review that has investigated partners’ social support in more detail, women interviewed emphasised the value they placed on the emotional and instrumental support provided by their partners. In interviews conducted with women who chose to terminate their pregnancy, the presence or lack of social support from partners was mentioned as a contributing factor to their decision. However, these findings have also highlighted women’s differing experiences with partners, as well as the complexity of partners’ social support and involvement in the Soweto context. While this and other current work to further explore women’s pregnancy experiences has focussed on women’s perspectives, the perspective of male partners has not yet been investigated. This study intended to address this gap in the research in order to provide a more complete picture of relational dynamics, and to understand male partners’ perceptions of their partners’ pregnancy and of fatherhood. Insights from this study can help to inform strategies for better engaging partners during pregnancy and encouraging greater involvement of fathers in their children’s upbringing, for the provision of nurturing care [22] to promote the health and development of their children.

## Methods

### Aim

The aim of this study was to qualitatively explore individual perceptions around relationship dynamics, their partner’s pregnancy, and fatherhood of partners of young women in Soweto, South Africa.

### Study design and setting

This descriptive study was embedded in the process evaluation of the *Bukhali* randomised controlled trial, which is being conducted as part of the Healthy Life Trajectories Initiative (HeLTI). HeLTI is an international consortium developed in partnership with the World Health Organization (WHO) in Canada, India, China, and South Africa. The *Bukhali* trial is being conducted with 18–28-year-old women in Soweto, [19] a densely populated, predominantly low-income, urban, and multilingual setting in Johannesburg. The intervention is delivered by trained community health workers, and is described in detail elsewhere [23]. The *Bukhali* process evaluation is focussing on context, implementation and mechanisms of impact, aligning with guidance from the UK MRC on process evaluation [24]. This context includes participants’ lived experiences, as well as their family, community, and social environment, [23] of which their partner and father of their baby (if they fall pregnant) are a critical component. Qualitative research methods are predominantly used in the *Bukhali* process evaluation in order to explore the complexities of context, implementation and mechanisms of impact. Previous qualitative research in the process evaluation have highlighted the important role of partners’ (fathers of the baby) social support for participants during pregnancy [25] and during decisions around termination of pregnancy [26].

For this study, individual, in-depth interviews were deemed to be the most appropriate method for exploring individual perceptions around relationship dynamics, their partner’s pregnancy, and fatherhood of partners. An inductive approach was taken to explore the data as per the research question posed.

### Participants

Male partners (fathers) of *Bukhali* participants were recruited for this study, with the agreement of *Bukhali* trial participants (female) to contact these participants being a prerequisite for recruitment. The female participant would need to be willing to provide contact details for her male partner, since there were no alternative ways in which to obtain the male partners’ contact details. This introduced an unavoidable bias in the sample and precluded the participation of men whose female partners were not willing for them to be contacted to be part of the study, or if the male partner was not available or was not interested in being part of the trial.

As part of the trial, if this agreement is provided, fathers are recruited during the pregnancy of their partner and invited for a range of biopsychosocial assessments. Recruitment for the interviews was done from the group who had completed or had indicated willingness to complete these assessments at the time of data collection for this study. From the list of fathers who had agreed to complete these assessments, 44 fathers were contacted who were still due to come in for these assessments. Of these, 27 were willing to participate in an interview, and 21 were able to schedule and attend the interview. Two of the interview recordings were unfortunately damaged, which left a final sample of 19 participants whose data were included in the analyses. Demographic details of participants are presented in Table 1, including the status of their relationship with the female trial participant, and whether they are cohabiting. Participants’ age ranged from 25 to 46 years (mean = 32.47 ± 6.08; median = 31).

### Interviews

Individual, in-depth interviews took place in-person at the research site from April–July 2022, and were conducted by a trained male interviewer and a note-taker who were conversant in local languages. A semi-structured interview guide was developed by the co-authors (available as supplementary material). These questions explored the relationship of the interviewee with their partner, the interviewee’s experience of pregnancy or during the pregnancy of their partner, their feelings about the support they provided to their partner, what they shared with or heard from their partner about the trial, and their thoughts on what could improve the participation of the fathers in the trial. The findings relating to their impressions about the trial and research in general are not included in this article. Interviews ranged in length between 20 and 78 min (average 37 min), were audio recorded, and were transcribed verbatim after being translated into English, where necessary.

The transcripts were quality-checked against recordings.

**Table 1:** Participants’ demographic details

Participant number	Age	Highest level of education	Employment status	Cohabiting
1	41	Grade 11	Employed	Yes
2	30	Matric* + certificate/course	Unemployed	No
3	32	Grade 11	Employed	Yes
4	36	Grade 12 + certificate	Unemployed	No
5	25	Matric	Employed	No
6	46	Matric + certificate/course	Unemployed	No
7	41	Grade 11	Unemployed	Yes
8	32	Grade 11	Unemployed	No
9	30	Matric	Employed	Yes
10	30	Matric	Employed	Yes
11	30	Diploma	Employed	No
12	36	Grade 10	Employed	No
13	29	Grade 11	Employed	Yes
14	29	Diploma	Employed	Yes
15	32	Matric	Employed	No
16	21	Matric	Unemployed	No
17	27	Matric	Unemployed	No
18	31	Matric	Employed	Yes
19	39	Matric	Employed	Yes

*Matric is equivalent of Grade 12, the final grade of secondary education in South Africa. †In a relationship with another woman.

### Data analysis

A thematic approach [27] was taken to the descriptive and exploratory process of analysis, starting with data familiarization and coding by MM. She generated initial codes inductively, staying close to the data and observing and searching for patterns across the transcripts [28]. The codes were broken down into subcodes by combining different codes to have overarching topics, which gave broader meaning to the patterns. MM generated a codebook from this list of codes, which informed the development of initial themes and subthemes by CED.

Quotes for each code were exported, and CED reviewed and refined the themes further in the process of collating and summarising participants’ responses. The analysis output was consolidated into three final themes and subthemes: (1) relationship dynamics (nature of relationship, relationship challenges); (2) pregnancy (feelings about the pregnancy, effect of the pregnancy on their relationship, providing support during pregnancy; and 3) fatherhood (view of fatherhood, roles of fathers, influences on views and motivation, challenges of fatherhood). These themes and subthemes are presented below, with selected illustrative quotes.

### Ethical considerations

#### Ethical approval

was obtained from the Human Research Ethics Committee (Medical) at the University of the Witwatersrand (Ref: M190449). All participants gave written informed consent for their involvement in the interviews (in addition to the trial). All procedures contributing to this work comply with the Helsinki Declaration of 1975, as revised in 2008. The *Bukhali* trial has been registered with the Pan African Clinical Trials Registry (https://pactr.samrc.ac.za; identifier: PACTR201903750173871, Registered 27/03/2019).

## Results

### Relationship dynamics

#### Nature of relationship

Around half of the participants reported to be living with their partner, but most described the current nature of their relationship with their partner as serious. The notion of ‘serious’ was mostly related to positive and healthy aspects of their relationship, such as good communication, commitment, trust, shared responsibility, learning from and understanding each other, and making each other happy. However, this was evidently something that changed over time, as a number of participants mentioned how the relationship had started as casual, but had become more serious over time, particularly as they got to know and grew closer to their partner. Some mentioned that their partner falling pregnant or having a child made their relationship more serious.

*“it was casual before she became pregnant…Because I saw the person she is. She made me happy in and out happy. And she gave me a child to express…So, that is why I’m saying it’s serious… Ja, it was a casual thing but as time goes by I thought about the future. I thought about now I’m getting old and I need to have a woman. I need to have a wife not a girlfriend.” (10)*.

*“It was a casual relationship and it became serious when uh about 6 months after because I wasn’t looking for somebody seriously I was just thinking about playing around but then I found that she was the one that I could spend my time with… we now have responsibilities, we have a child, and I like how uh she makes me mature like her advice that she gives me like she shows me that life is like this and that so that’s mostly the things that I like about her.” (12)*.

A few participants described their relationship as casual, which for some began in this way due to them or their partner recently coming out of another relationship. In terms of what is defined as ‘casual’, one participant’s response highlighted the cultural understandings of this in terms of relationships. A few other participants said that they were no longer in a relationship with their partner, or that the relationship was not going well.

#### Relationship challenges

Participants mentioned a range of challenges that they experience in their relationship with their partners; many spoke about how they are working through challenges, and asking others for help and advice. Challenges included differences in thinking and what they want, not agreeing with each other, not being honest with each other, and a lack of communication. A few participants mentioned other challenges such as their partner focussing on her friends too much, problematic drinking habits, and unfaithfulness. Some participants were experiencing challenges in their relationship as a result of having their partner’s family’s approval for their relationship or the pregnancy.

*“sometimes we don’t think the same way, number two we don’t want the same things, number three to make someone understand what you want and understand them as a person to understand what they want; because we want different things along the way.” (1)*.

*“It was as I’m saying like lack of communication, like lack of making time because she was always busy, I was always busy like you know.” (9)*.

*“challenges that I have faced is that [name] would feel left out or lonely in a way because she would call at work during the day while I’m at work and she would cry, I don’t know if she was frustrated by the fact that the baby is also crying and she can’t make her stop crying or if she was feeling like her life is over in a way.” (15)*.

For two participants, the challenges related to losing their baby, which brought about a change in their relationship dynamic. For another participant, it seemed as if his partner had experienced post-natal depressive symptoms.

*“she fell pregnant which she had a miscarriage right. Yes, so there was a distance between us, it was no longer the same as we started the relationship.” (4)*.

### Pregnancy

#### Feelings about the pregnancy

Many participants stated that they only found out their partner was pregnant was when she missed her period. Most participants reported that their partner’s pregnancy was not planned; only three participants specifically mentioned that the pregnancy was planned, while some were not clear. Many participants expressed feeling positive about the news of their partner’s pregnancy, which included feeling happy or excited due to their desire to have children or loving children.

*“we’ve been looking to have a child after all that happened…I was happy my brother, I was very happy, I was happy and I was even coming home from work so I was like today let me skip gym and rather spend time with them.” (14)*.

Participants frequently mentioned having mixed feelings when they found out their partner was pregnant. These included feeling happy and excited, but also feeling shocked, disappointed, hesitant, nervous, scared, and stressed. These less positive emotions appeared to be linked to cultural expectations, perceptions of how family members would react, or not feeling ready for a baby; one participant commented on the complexity of being ready. It was evident that these participants understood the level of responsibility that was expected of them as a father. One participant was initially shocked, but came to accept the pregnancy without expressing clear feelings either way: “*it is what it is*” (13).

*“when I found out that my girlfriend is pregnant I was happy, at the same time nervous because I knew what was going to come as a soon as they find out that this person is pregnant…I don’t think there’s anyone that is ever ready, once you become a father it means you’re ready; so there’s no right time to say you’re ready now or whatever; so I’m always ready.” (1)*.

*“I was happy, like totally I was happy but at the very same time I was scared because I was thinking like how am I going to support the child.” (16)*.

A common feeling amongst participants was concern about how they will provide for the child and mother, particularly for those who were not employed – either the father or the mother, or both of them. Future aspirations involved being employed and able to provide for their family; for some, these aspirations were not being met and this had caused challenges in their relationship.

*“I also want to get full time employment so I can work for her as her man, ja….We’d just start arguing about the child and how we’re both unemployed with a baby on the way and how expensive diapers are, like when we go to the shops to buy food she’d just lose it over how expensive diapers are and that would stress me out even more especially when we were both still out of work, she’d just freak out that the diapers are expensive and clothes are expensive.” (3)*.

*“I used to also contribute at home while also maintaining my child; but now that I don’t work, I have nothing you know so I have to hustle where I can and then be able to provide for both my children so they are taken care of so their mothers don’t think I’m useless you understand, sure.” (17)*.

One participant shared in more detail about his mixed feelings, specifically his initial shock and feelings of not being ready, considerations about terminating the pregnancy, and also contemplating his responsibility as the father of the baby.

*“Eish I felt like I was going to die because I wasn’t ready for another baby, honestly she was, we were not ready for another baby…Ja, and then I feel like I just honestly I didn’t want the baby…I would get suggestions of getting rid of the baby like abortion you know but I’m like, so if you are going to abort this child and honestly you knew that you got her pregnant. If you are going to abort this child, how are you going to live with yourself, remember you have done something to yourself and what about, what if she dies, you know what if there are complications, you understand. What if she doesn’t give birth anymore and all because of your selfish ways? All because you didn’t want to be responsible, you didn’t want to be responsible from the first place, from get go you guys having unprotected sex, to you impregnating her.” (9)*.

### Effect of the pregnancy on their relationship

Some participants believed the pregnancy had a positive effect on them, and on their relationship with their partner, such as bringing them closer or encouraging them to be more serious about their relationship. A few other participants mentioned the effect of their partner’s pregnancy in a less positive light, including not being able to do the same things as before, such as watching sport; or that they felt guilty because their partner was feeling sick, or they actually became sick as well. Two participants spoke about the challenges they experienced trying to balance their attention between their partner and their baby, if the baby had been born.

*“But positively it affected me in a sense that I became happy mostly knowing that I was expecting a boy child.” (6)*.

*“Yoh pregnancy symptoms my brother, like it affects her and then in turn I get affected; because she throws up and I do too.” (14)*.

*“Ja I’d say that I think I became more selfish towards the child, like things that changed is that I just focused on the baby more than anything else; I thought she understood why but only to find out no.” (11)*.

Some participants had experienced more negative effects of the pregnancy on their relationship in terms of how the pregnancy had caused changes or challenges, such as fighting more, or experiencing frustration with each other. Part of these relational challenges seemed to be the difficulty participants experienced dealing with their partner’s emotions, such as annoyance, as well as moodiness and changes in moods. For those experiencing a partner’s pregnancy for the first time, these challenges seemed to be particularly difficult.

*“Okay uh at some point it was frustrating to deal with a pregnant person because it was the first time for me, and it came with a lot of challenges.” (15)*.

*“eish it was an experience that I’ve never went through. I didn’t know how to take care of a woman who is pregnant. But coming here ja, then I was, I was aware of how to take care of a pregnant woman. They taught me how to take care of her emotions. And not to stress her, ja.” (10)*.

*“I was short tempered with her at a point and then end up not picking up her phone calls cause I thought maybe she was making a fool out of me because she would make me leave the house like maybe at night and say her tummy is sore and that I must come rub it and then only to find that when I get there she’s fine and all she wants is to spend a bit of time with me at home.” (17)*.

### Providing support during pregnancy

With regards to the support they provide their partner during pregnancy, some participants described in general terms, such as “*supportive in every way, every way…just be there, be present, be supportive*” (9), whereas others acknowledged that support has various components: “*Be supportive, financially, emotionally, like always be there for both the mother and the child*” (11), “*I would advise them to be medically involved, emotionally involved, even financially involved*” (6). Numerous participants acknowledged that support entailed being present and available for their partner, which also practically involved taking time off work, communicating regularly, attending check-ups and ultrasound scans. One participant mentioned supporting her participation in the trial.

*“I did attend anti-natal visits. Each time she went for a scan or she went to the clinic I accompany her.” (2)*.

*“I tried my utmost best to support her in any way I can because I’m always at work and the two days I get off I make sure that I’m with her and I try calling her from work so that she doesn’t feel unwanted or left alone during this whole thing of pregnancy you know, so I think I did my best to support her.” (15)*.

*“she also told me that there’s this study from here and I said you know what let me support her because if I don’t support her it’s going to end up badly and we’ll blame each other, so I said no let me go and see for myself and then I became interested in how they do things here.” (8)*.

The provision of emotional and social support during pregnancy was frequently mentioned by participants, and this involved showing love, trying to relieve stress (or not cause stress), spending time together, and making sure their partner is happy and not upsetting her.

*“what do fathers do ne, the father like during the pregnancy they must like take care of their girlfriends while they are pregnant, not arguing too much, each and every time you need to be calm because whatever argument you are having, it can affect the child.” (4)*.

*“during pregnancy you need to calm down, you calm down because like your women she is going to go through like stress.” (10)*.

*“But being there for her come, it gave her strength that through pregnancy, through the pains that she went through things became easier because I was there to encourage her and support… But being there for her come, it gave her strength that through pregnancy, through the pains that she went through things became easier because I was there to encourage her and support.” (7)*.

For some participants, showing support entailed providing the resources needed by their partner, such as financial resources, food, clothes, and blankets for the baby. There were other practical ways in which participants talked about showing support, such as helping her with whatever she needs, being involved in her pregnancy care, and making sure she is comfortable. For some participants, this practical support seemed to have a protective element in terms of reducing risks, and making sure their partner is receiving the care needed for her and the baby.

*“so even when she wanted something I would buy it for her, sometimes we’d leave the house and go have fun at the malls.” (8)*.

*“in terms of parenting or in terms of taking the child for immunisation what, what I feel like changing nappies aha bro this is a 50/50 thing. It is a 50/50 thing me sometimes I make it 60/40 thing you know because sometimes I feel that she is tired she has got stretched you know she has had enough you understand, she has been carrying my son for 9 months man, like that is the least that I can do change nappies.” (9)*.

*“when she goes to the clinic uh like I used to get into the queue for her like early in the morning and then she’d join the queue during the day when I had already been in the queue, and just give her support for whatever she wants.” (13)*.

It was evident from some participants’ comments that there had been changes in the social and/or cultural expectations of fathers. Other cultural expectations appeared to be persisting, such as the order in which daughters should be married, and the payment of ‘*lobola*’ (bride price). Some of these related to cultural norms that influenced the involvement of the baby’s father, particularly if the couple have not gone through traditional rites around marriage, including *lobola*.

*“I can also include culture there to say most of us know that men don’t look after the child, a man doesn’t cook or do laundry; no that thing is no longer relevant; we are going 50/50 now guys.” (3)*.

*“But [name] comes from a Venda and Sesotho family background. So, the cultural way they do clash but they are very routed in their African belief that if a man has not yet paid lobola cannot just come into the yard and go out what we call in our kasi lingo to come and ‘check’.” (6)*.

*“It’s about culture the problem. She is a Pedi, I’m a Xhosa. Now the Pedi if are two girls because she is the last born, if they are the two girls from before her, their culture says the last born can’t get married before the others do. So, now that’s the problem that we are facing, and that is the challenge my family are saying as well that now should the last born wait for the other to get married or what.” (7)*.

A number of participants mentioned the COVID-19 pandemic in relation to barriers to providing support, as they mentioned that clinics did not allow partners to come in for consultations. Linked to this, one participant’s comment indicated that health facilities do not necessarily encourage the presence of the father; another mentioned that he was not always able to go to these visits due to other commitments. Lack of financial resources as a result of un- or under-employment was another barrier to providing the type of support mentioned, which relates to concerns about being able to provide. This challenge extends beyond pregnancy, and is discussed further below.

*“I accompany her, but since it’s been with Corona virus there is a lot of, also we are not allowed to go, she goes in alone. I take her there and when she is done she calls me and I fetch her.” (2)*.

*“I’m a young guy, I’m not supposed to, I’ll rock up there and be like all nicely dressed up at the clinic because I love clothes; and they’d be like. What is happening with this guy you understand get different looks, but now I don’t care you understand, I’m here to get my child immunised and then go.” (9)*.

*“maybe you find that sometimes I don’t have money, I had to hustle at home or tell my uncles that eish man this is the situation please can you borrow me money and then I’ll pay you back, and then you find that we don’t get it and then we end up trying loan sharks to borrow us even R50 so she can go to the clinic and get her check-ups done.” (8)*.

### Fatherhood

#### Views about fatherhood

Participants’ views about fatherhood were overwhelmingly positive. Feelings of happiness and fulfilment stemmed from just being happy to be called “daddy”, being able to be present for their child and there when they need them, feeling blessed or grateful to see their child grow up, leaving behind a legacy, the sense of pride felt when their child wants their attention and look to him for help, guiding and advising their child, and contributing positively to their child’s future and to the person they will become. Some participants spoke about the love they have experienced (given and received), which is sometimes felt even in the practical things they do to help. Others spoke positively about being responsible for and taking care of their child.

*“I feel good, when I wake up and there is someone calling me daddy, daddy it makes me feel happy.” (19)*.

*“Yes, there are like it feels good like to live knowing that there is someone who like depends on you and who needs you, to be there and to guide the child and make sure there is nothing that the child suffers for or needs…Having someone call you like dad, and that like there is that like respect, that feeling of like being a dad. Somewhere, somehow like spiritually you feel grown…Like deep down like I don’t know how I can put it but there is this feeling that makes you to like never give up in life, or never accept failure because there’s always that bond you have with your child or family, and ja it comes from deep down and nothing can break it, like it is a stronger bond.” (2)*.

*“It’s supporting my kids and seeing them grow up and for them having a bright future…Like you also get excited that at least in life uh I’ve brought someone into the world, like you get happy seeing them like you want to see them being somewhere in life one day.” (13)*.

### Challenges of fatherhood

For many participants, the challenging aspect of being a father related to having to provide financially, especially if they were not employed, and making their child their financial priority.

*“Challenges of being a father my man is that you must hustle, you need to work for your child; not to say they shouldn’t go without but it shouldn’t be that every time the child needs something then we say we don’t have; you must try by all means to make sure the baby goes to bed fed; like now the challenge is that she’s ready to stop breastfeeding so the next challenge is the next step that she must get formula, that is a challenge you know, even having to dress the baby knowing that you don’t work, it’s a challenge.” (1)*.

*“the most challenging part of being a father is okay, I check it from my friends, ja those ones who are not working right now, it’s like it’s a little bit tough because there is no like small income coming in, like let’s say maybe if you had a small shop or selling some things like they are depending on their family, like their uncles, you know.” (5)*.

At times, the prioritisation of their child appeared to come at the expense of their partner for some participants. For some, this was in terms of finances, whereas for others it was about time spent with their baby rather than their partner. However, one participant specifically mentioned this focus on his baby alongside ensuring that his partner was happy and provided for.

*“It’s just fine because what I’m concentrating on is the baby, that is the very main concern, is my baby you understand…but now I just want us to focus on the baby to make sure that the baby is not short of anything…I just want us to focus on the baby and make sure that everything is fine for the baby. And as I said that she must be happy. I’m saying in such a way that I’m making sure that the child has everything, ja that she doesn’t have to struggle, and each and every time I have to phone, like you have to buy milk what, what you see that is what I’m saying.” (5)*.

Other challenges mentioned by participants included the high use of diapers, sleepless nights, concerns they have related to ensuring their child’s wellbeing, and not living with the mother and hence not seeing their child every day.

*“The most challenging part is the diapers…I’ve learned that a new born, eish she wastes diapers like nobody’s business hey.” (7)*.

*“most challenging part is that I would say like at this stage where the child is a bit too small she can’t speak for herself…that can be quite challenging because you are not really sure what the problems is.” (18)*.

### Roles of fathers

In terms of what participants perceived to be the roles of fathers, some articulated this from a broader perspective in terms of being responsible, using terms such as “man up”, “leaders in the house”, and “oversee”. Others referred to the necessary shift in their lifestyle and priorities in acknowledgement of this increased responsibility. A few participants saw this sense of responsibility in more practical terms, and saw their role as being hands-on and taking on some of the caregiving responsibilities in order to share these with their partner.

*“I can just say since she told me that you know what she is pregnant, that is when I saw that you know what this is time now, that maybe we need to man up now. You are growing up now you are going to have a child, you need to start being responsible, to change lifestyle…I can say it’s nice being a father it is like, it’s giving you how can I put this, like to be responsible…I was a boy now I’m a man.” (5)*.

*“I was someone that liked hanging out with the guys, you know like we’d go out with the guys and then it ended up with me saying guys things are going to change with me with a lot of things; I’m no longer going to gallivant around and play around because I now have two kids so I need to start looking out for things in the house and what is needed there you understand.” (13)*.

*“Uh my role is the same as the mother it’s just that I don’t have a breast to breastfeed her but it’s all the same; I have to change diapers, carry the baby, go to clinics, each and every thing that the mother is supposed to do I’m supposed to do too, it’s not her responsibility alone it’s for both of us.” (12)*.

In terms of other more specific roles, it was clear from participants’ responses that there were many, and this included providing for their partner and child, which echoes to what was discussed earlier about provision as part of showing support, as well as some participants’ concerns about how they would be able to provide. Participants also talked about their role as a father as being supportive, active, present, involved, and taking care. A few participants mentioned their role of protector and guide for their children, and one participant acknowledged the sacrifices that they needed to make.

*“…role as a father is to be there for the both of them, all the times, ja, like I feel like I had to be the first one even when the baby was born so I could see her, ja, support them, give them support all the times…My role as a father is to provide for the child, make sure the child is growing well, healthy and okay.” (16)*.

*“Eish, a father’s role should be all those things, you need to be supporting and active; yes you need to be supportive and active like all the time….Even after she’s breastfed you need to take the baby and burp them, you know she needs to feel your comfort, the baby must feel that okay this is my father; because if you miss that stage, it’s over my man.” (1)*.

*“Fathers should protect them and guide the kids as well and take care of them financially spoil when they are still small…support them in everything that they want to do but also guide them make sure it is the correct path that you can also see that something good that you want to go into. So, guidance should be the key role as well.” (18)*.

Some participants expanded on their description of what it meant to be a present and/or involved father, and their responses centred mainly around love and connection. Their descriptions included providing for their partner and child; being with their child when they are sick; spending time together; bonding with their child and getting to know them and they get to know you; being loyal, committed and loving to their partner and prioritizing her happiness; and showing love and respect for each other.

*“….even when the child is sick, I am able to take a day off, I just call them at work and tell them that my child is not well, Because when my child do not feel well I also don’t feel well, the way I love my child.” (19)*.

*“it means a lot like I need to be there for her during this whole process. I need to give her my support, she is not alone in this I’m with her. So, I need to show her that support to you know what we are together in this and we are going to make it.” (4)*.

On this topic of involvement of fathers, two participants commented on how not all fathers are given this opportunity, suggesting that partners (and/or their families) may be gatekeepers for fathers’ involvement in their children’s lives. In some cases, participants mentioned that this was due to them not paying *lobola*.

*“I’ve discovered that many men are not there for their children of which I don’t blame them. I don’t know the conditions of them and everything. But that is what I’ve noticed that most women they go through pregnancy issues alone. At some point again we fathers as well we can’t bring men at all times, but even women sometimes they close channels for us to partake in the issue of pregnancy. Or in the issue of being there for our children. So, I think fathers should always be given that opportunity to take decision if they want to be there or not. Then it will make life easier.” (7)*.

### Influences on views and motivation

Participants’ provided insight into what has influenced their views on fatherhood and their motivation to be present and involved in their children’s lives. For numerous participants, their positive views on fatherhood and roles stemmed from their own experience of growing up without a father (and sometimes mother), and not wanting their children to experience the same. Some of these participants specifically referred to the pain of this experience and the mistakes of their father, while for others this was more implied. One participant added that his Christian belief about the importance of family added to his motivation to be present for his children.

*“I grew up without a mother and a father, my brothers raised me, they took me to school. So, I would like to see my children grow up with my own eyes.” (19)*.

*“I grew up without a father actually, so I can say that it’s something that I told myself that I don’t want my child to experience the same things that I experienced so that is why I managed to make sure I’m always there for her.” (16)*.

Conversely, other participants were motivated by the positive role played by their father, who they felt was present, committed to their family, and provided for them. Participants also mentioned other men in their community, including other family members, being positive role models of fathers, or even being motivated by the positive portrayal of fatherhood in movies.

*“I’m getting it [motivation] from my father. My father has been present for like I lost my mother when I was doing like grade 6. Ja, then my father was my sole like taking care of your children as he parented me…he cooked for me, he washed me, he bathed me. So, he taught me how to take care of a child, even now he is still teaching me as I’m a parent.” (10)*.

*“so I have two cousins at home right, they love their children so much so, that I believe that I love my child the way they love their children, and it’s encouraging to see them enjoying the fact that they are fathers.” (15)*.

Participants’ responses suggest that there has been something of a generational shift in norms around fatherhood, and that being present and involved is more common in the current generation of fathers. However, the examples given above of good fathers in the previous generation, as well as participants comments about some men still abandoning their children suggest that norms around fatherhood are mixed and complicated in this context.

*“none of them ran away from their babies, we’re always there and seeing another man doing what we’re doing motivates us, because we do it… even everybody in my neighbourhood they say you’re a great father because you are always there, you take the child to school and all that, so it teaches other guys what you’re supposed to do, ja… father’s side right he had other kids in Pretoria so my relationship with him wasn’t real or what because him and I saw each other every second or third time in a year; so we didn’t have much. So I didn’t want that to pass onto my kids, so that is why I give them the much love that my daddy didn’t give me, yeah.” (12)*.

*“okay there is some fathers in our community but in the township honestly you don’t get that positive influence because most of the uncles there they are not there for their children you understand.” (9)*.

## Discussion

The findings of this study have highlighted unique perspectives of male partners of participants in a trial such as *Bukhali*, which helps to address the general absence of men, and specifically fathers, in research on maternal and child health [3]. Given that previous process evaluation work with *Bukhali* has not included these perspectives up until now and only reported on female participants’ perspective of relational dynamics with partners, these findings help contribute to a more complete picture of the lived experiences of the female trial participants.

A key finding of this study is that amongst the men who participated, their views about fatherhood were overwhelmingly positive. This was within a context of largely unplanned pregnancies; although participants’ comments suggest that fatherhood may be viewed as a somewhat expected and inevitable life event for these men, and for some participants becoming a father seemed aspirational. These findings confirm the complicated notion of a father being absent yet involved, [4, 7, 8, 29] since less than half of these fathers resided with their partner and child, but almost all characterised their relationship with their partner as serious, drawing on positive aspects of their relationship to substantiate this characterisation. Further to this, there was the sense that participants recognised the importance of love and connection with their child/children as part of their presence and involvement, which is encouraging given the importance of responsive caregiving for early childhood development, [22] and the positive impact of the father-child relationship on early childhood development outcomes in low- and middle-income country (LMIC) settings [30, 31]. In this study, participants’ responses highlighted the complexity of fathers’ presence and involvement, and the range of positive and negative life experiences (e.g., positive role models, or their own absent father) that influence these men’s motivation to be present and involved fathers, which aligns with previous research on fatherhood in South Africa [7]. While the reality of the high numbers of children in South Africa growing up without the positive influence of a father should not be disregarded, participants’ perspectives indicate that there is possibility and desire for a change in narrative around fatherhood in a context such as Soweto. These shifts have been the topic of previous research that highlighted perspectives of South African men, and how they incorporated fatherhood into their identity, and sought ways in which to alter the narrative of fatherhood that they had experienced [29].

Participants also seemed to have a well-rounded understanding of the need for providing support to their partner and child. This included emotional and social support, as well as more practical, ‘hands on’ support associated with childcare, with some noting how men are playing a more active role in this type of practical support. The structural barriers within the South African public health system that hinder the involvement of fathers [9] need to be addressed to further facilitate their support of mothers during pregnancy and birth. These findings regarding support align with research mentioned previously regarding the shifts in gendered caregiving roles in South Africa [4–6, 15] and other African countries [16, 17]. Furthermore, these findings indicate the need for further work to explore understandings of father involvement, drawing on contextual realities rather than Westernised notions of involvement [18]. However, the shifting – and stability – of caregiving roles needs further investigation in a context such as Soweto, given that caregiving as women’s responsibility is known to have been strongly influenced by cultural and gender norms in other LMIC settings [18, 32, 33].

Another key finding of this study is that within the context of largely acknowledging the weight of responsibility that accompanies fatherhood, participants viewed providing for the material needs of their partner and child as a key role of being a father. This supports previous research findings regarding fathers playing a traditional role as financial provider [29, 34, 35]. However, this role of financial provision was also framed as one of participants’ main concerns in this study, and the lack of financial resources was seen to be a barrier to providing support to their partner and child; this was particularly concerning for participants who were not employed. It is possible that concerns about providing financially are worth exploring further as a potential reason why men in a context such as Soweto might not cohabit with their partner and child, even when they are in a committed relationship.

Another potential reason for not cohabiting could be the challenging family dynamics mentioned by some participants, which have been identified in previous HeLTI research [26, 36]. For this study, these family dynamics were particularly salient in terms of the social and cultural expectations of men relating to parenting and marriage, but also relevant for family members’ approval of male partners, the payment of ‘*lobola*’, and the extent to which family members may act as gatekeepers of fathers’ involvement in their children’s lives. The role of cultural gatekeeping in determining father-child involvement has been highlighted in research from a rural South African setting, particularly for unmarried fathers who do not live with their partner and child [37]. ‘*Lobola’* remains a contested but changing cultural practice in South Africa, [38] although it is often not affordable for men with challenging economic circumstances, [39] such as the men in this study. Previous research in South Africa has explored other practices of non-marital cohabiting, but in some settings these are frowned upon [39, 40]. These factors likely add to the complexity of changing fatherhood narratives in the Soweto context, with some gendered roles shifting, while other more ‘traditional’ views prevailing that do not necessarily support these shifts. Other factors that could potentially influence changing gender roles include local and global discourses on inclusion and gender equity, particularly in younger generations. In South Africa, efforts to promote gender equity and father involvement are being increasingly supported at policy, media, and educational campaigns. While these changes may not be uniform across all settings in South Africa, further research is needed to track the impact and sustainability of these efforts. Furthermore, the economic pressures faced by these men could interact with cultural expectations, in the sense that some men might find it financially difficult to pay *lobola*, which could affect access to their child.

As mentioned previously, given that trial participants had to agree for their male partner to be contacted, the sample of male participants represents a somewhat unique sample of men who are at least present in their partner’s life and on good enough terms for their partner to be willing for them to be involved in the trial. It is therefore possible that men who fit into this category would be more positively inclined to fatherhood, and may present more positive views; they may have attempted to present themselves in a more positive light, considering the narratives around absent fathers in South Africa. Although this may be viewed as a limitation of this study, the descriptive nature of this study nevertheless captures the views of these men. Capturing the views of absent male partners (who may not even agree to an interview), or those who female participants did not agree to be contacted would be extremely difficult, if not impossible.

In conclusion, the study findings suggest that the complex array of factors influencing fatherhood in South Africa [4–6, 10] continue for this generation, although there are promising shifts. Exploratory qualitative research plays a valuable role in capturing these complexities and changes in a context such as Soweto, and it is critical that these are better understood especially when intervening through a trial such as *Bukhali* with young women and their children whose lives are affected by their male partners and fathers respectively. Furthermore, it is imperative that these complexities and changes are considered when planning and implementing other interventions to promote involved fatherhood in South Africa. Such interventions should also draw on the notions of love and connection between fathers and children, and provide guidance for men to navigate the provision of material support in the context of economic challenges. The insights gained from this study can help to guide future research with men both in and beyond Soweto to further explore the complexity of fatherhood in South Africa, and to develop and evaluate contextually relevant strategies to further shift narratives in a positive direction.

## Data Availability

Data can be made on reasonable request to the corresponding author.
